# Co‐Infection by *Porphyromonas gingivalis* and *Fusobacterium nucleatum* Increases PD‐L1 Expression in Esophageal Cancer Tissues

**DOI:** 10.1002/mbo3.70050

**Published:** 2025-08-11

**Authors:** Xiayi Lin, Xiaohui Chen, Mengyuan Chen, Yiqiu Li, Yu Deng, Bohong Xian, Zijun Li

**Affiliations:** ^1^ Department of General Practice, Guangdong Provincial People's Hospital (Guangdong Academy of Medical Sciences) Southern Medical University Guangzhou China; ^2^ Department of Gastroenterology Puning People's Hospital Puning Guangdong China; ^3^ Department of Microbial and Biochemical Pharmacy, School of Pharmaceutical Sciences Sun Yat‐sen University Guangzhou China; ^4^ Institute of Systems and Physical Biology Shenzhen Bay Laboratory Shenzhen China; ^5^ Department of Gastroenterology Boai Hospital Zhongshan Zhongshan China

**Keywords:** esophageal cancer, immune escape, PD‐L1, tissue microbiology, tumor microenvironment

## Abstract

Currently, the impact of the microbiome on the progression and recurrence of esophageal cancer remains unclear. Microbes have been demonstrated to increase programmed death‐ligand 1 (PD‐L1) expression in certain epithelial tumors, thereby facilitating immune escape. Therefore, we hypothesized that the microbiome in esophageal tissues might be involved in the progression and recurrence of esophageal squamous cell carcinoma (ESCC) through PD‐L1 upregulation. To assess this possibility, we analyzed the relationship between the microbial community composition and PD‐L1 expression. We found that the microbiome of esophageal cancer tissue significantly differs from that of normal tissue, and *Porphyromonas gingivalis* and *Fusobacterium nucleatum* could represent markers of ESCC. An immunohistochemical assay revealed that *P. gingivalis* and *F. nucleatum* infection led to a significant increase in PD‐L1 expression on the membrane surface of tumor cells, and this finding was further confirmed in cell experiments. Our findings suggest that eliminating intratumoral *P. gingivalis* and *F. nucleatum* infection could represent an important therapeutic strategy for reducing the progression and recurrence of esophageal cancer.

## Graphical abstract

*Porphyromonas gingivalis* and *Fusobacterium nucleatum* (Fn) co‐infection increases PD‐L1 expression in esophageal cancer, aiding immune escape; eliminating them may reduce progression and recurrence.

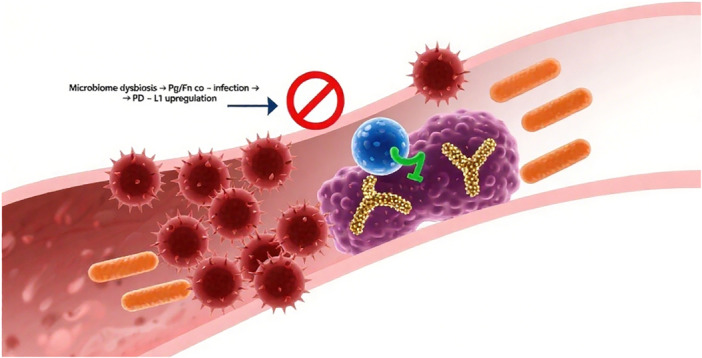

## 1 Introduction

Esophageal cancer, a serious and malignant tumor, ranks seventh in global cancer incidence and sixth in cancer‐related mortality. Esophageal cancer can be classified as squamous cell carcinoma or adenocarcinoma based on its histopathological features. Esophageal cancer is the main cancer type in Asia (Lagergren et al. 2017; Sung et al. 2021). The conventional treatment of esophageal squamous cell carcinoma (ESCC) includes surgery, chemotherapy, and radiotherapy (Cowie et al. 2014; Ferlay et al. 2015). Although the most common treatment for esophageal cancer is surgical resection, some patients cannot undergo surgical resection. In particular, the R0 resection rate among patients with locally advanced esophageal cancer is approximately 50%, and the rates of disease progression and recurrence are extremely high (Medical Research Council Oesophageal Cancer Working Party 2002). Therefore, clarifying the mechanisms of progression and recurrence of esophageal cancer is crucial for guiding subsequent treatment. Researchers have extensively explored the causes of progressive recurrence in ESCC, but the precise causes and mechanisms remain elusive.

To date, several studies have demonstrated that immune evasion can promote the recurrence and aggressive progression of esophageal cancer through activation of the programmed death‐ligand 1 (PD‐L1)/programmed death receptor 1 (PD‐1) signaling pathway (Okadome et al. 2020; Ohigashi et al. 2005). As a member of the B7 costimulatory molecule family, PD‐L1 (B7‐H1) mainly binds to PD‐1 expressed on the surface of T and B cells, thereby promoting cell apoptosis, weakening host immune function, and facilitating immune escape by tumors (Blank et al. 2005; Shi et al. 2013). Okadome et al. (2020) reported that the recurrence and progression rates of PD‐L1–positive ESCC are higher than those of PD‐L1–negative ESCC, indicating that PD‐L1 expression promotes ESCC recurrence. PD‐L1 expression is regulated through the complex interplay of multiple factors and mechanisms, including IFN‐γ and the microbiota (Cha et al. 2019; Sun et al. 2018; Tang et al. 2018; Groeger et al. 2011, 2017; Gollwitzer et al. 2014). The microbiota have been demonstrated to increase PD‐L1 expression and facilitate immune escape or immune tolerance in specific cases of epithelial tumors and allergic diseases (Groeger et al. 2011, 2017; Gollwitzer et al. 2014). However, the effect of the microbiota on PD‐L1 expression in ESCC tissue remains unknown.

Chronic infections promote cancer developments (Xia et al. 2023). In a global study, researchers discovered that approximately 18% of the global cancer burden could be attributed to pathogen infections (Bornschein and Malfertheiner 2014). Specific microbial imbalances exert carcinogenic effects. For instance, *Helicobacter pylori* is currently the most well‐established carcinogenic bacterium, as it can destroy the gastric mucosal barrier, promote CagA release, activate the NF‐κB pathway to promote chronic inflammation, and induce the aggregation of mesenchymal stem cells, among other mechanisms, to facilitate the development of gastric cancer (Dutta et al. 2000). Recent studies on *Porphyromonas gingivalis* in oral head and facial tumors also revealed its role in carcinogenesis. Using a model of 4‐nitroquinoline‐1‐oxide‐induced oral squamous carcinoma, researchers found that *P. gingivalis* infection stimulates toll‐like receptors (TLRs) in oral epithelial cells, thereby activating the interleukin (IL)‐6/STAT3 signaling pathway and promoting the growth of oral squamous cell carcinoma (Mima et al. 2015). Masugi et al. discovered that *Fusobacterium nucleatum* could regulate the distribution of T cells and the expression of PD‐L1 in colorectal cancer tissues, thus affecting tumor development (Masugi et al. 2017; Yu et al. 2010). Additionally, microecological imbalance can promote carcinogenesis. Despite the absence of microorganisms in the liver, studies reported that intestinal microecological imbalance promotes the development of liver cancer. This finding might be attributable to the fact that intestinal bacteria reach the liver through the portal vein by releasing inflammatory factors such as endotoxin and bacterial metabolites, causing chronic liver damage, inflammation, and fibrosis, which ultimately facilitate liver cancer (Ochi et al. 2012). Recent studies indicated that the intestinal microbiota can release virulence factors such as lipopolysaccharide to activate chronic inflammation of the pancreas and promote pancreatic cancer (Geller et al. 2017). In conclusion, increasing evidence indicates that intra‐tissue microorganisms may be associated with tumorigenesis. The GI tract has the largest and most complex variety of microorganisms in humans, and thus, GI tumors are perhaps the most strongly related to alterations in tissue microecology. Therefore, the gastrointestinal microbiome is both the most influential microbiome concerning human health and metabolic status and a useful model for studying microbiota–host interrelationships and disease occurrence (Pei et al. 2004). Based on the potential correlation between the microflora and tumor, we propose that the microflora in ESCC tissue can affect the recurrence and development of ESCC by increasing PD‐L1 expression.

Our findings demonstrated notable variations in the abundance of *F. nucleatum* and *P. gingivalis* between patients with ESCC and controls, suggesting that these microorganisms can serve as markers of ESCC. We further verified the relationship between the microbiota and PD‐L1 expression in cell experiments to determine the role of the microbiota in tumor recurrence.

## 2 Methods

### 2.1 Study Participants

Patients with ESCC who underwent thoracoscopic partial esophagectomy or gastroscopy at Guangdong Provincial People's Hospital from January 2014 to December 2015 were screened according to the following inclusion criteria: age of 18 years or older; histopathological and diagnostic evidence of ESCC; good overall health status with no evidence of metabolic disorders such as diabetes or hyperlipidemia and no indications of infectious diseases; no receipt of antibiotics, acid‐suppressive agents, or probiotics affecting esophageal microbiota composition within 2 months of enrollment; no special dietary habits, such as the consumption of betel nut; and no serious liver or kidney dysfunction or immune deficiency. Meanwhile, the exclusion criteria were as follows: use of drugs that affect esophageal microecology within 2 months before enrollment; diagnosis of autoimmune diseases; diagnosis of cancers other than esophageal cancer; and incomplete clinical data. A cohort of 30 patients was recruited for this study, and pathological samples were collected from the participants. In addition, 25 healthy individuals with no abnormal results on gastroscopy performed at Guangdong Provincial People's Hospital were included as healthy controls (the body mass index of all healthy individuals was within the normal range). The research protocol received ethical approval from the Ethics Committee at Guangdong Provincial People's Hospital, and all participants provided written informed consent before enrollment.

### 2.2 Collection of Specimens

Esophageal cancer tissue samples were obtained from the center of the cancerous lesion and fixed in neutral buffered formalin solution. The samples were then paraffin‐embedded and consecutively sliced into five sections with a thickness of 4 µm, and the sections were placed on slides coated with an anti‐detachment agent. All data were obtained in the Department of Thoracic Surgery and Pathology. For healthy controls, two biopsies were taken from the normal mid‐esophageal mucosa under endoscopic observation. The biopsies were fixed in neutral buffered formalin solution and immediately stored at −80°C for preservation.

### 2.3 DNA Extraction

The tissue samples were eluted, and suspensions were generated using 1 mL of phosphate‐buffered saline (PBS, pH 7.4). These suspensions were centrifuged at 3000 ×* g* for 5 min, after which the supernatant was carefully discarded. The pellet was subsequently resuspended in 200 µL of PBS and 20 µL of protease. DNA was extracted from saliva samples using the UltraClean Microbial DNA Isolation Kit (MOBI, USA). The NanoDrop One system (Thermo Fisher Scientific, Waltham, MA, USA) was used to assess the concentration and purity of the extracted DNA. The filtrate was then stored at −20°C.

### 2.4 Sequencing Data Processing

The prokaryotic primers F515 (5′‐GTGCCAGCMGCCGCGGTAA‐3′) and R806 (5′‐GGACTACVSGGGTATCTAT‐3′) were used to amplify the 16S rRNA genes of bacteria by combining the sample‐specific 12‐base barcode on F515. The amplification protocol consisted of a preliminary configuration phase of 5 min at 94°C; cycles of denaturation at 94°C for 30 s, annealing at 52°C for 30 s, and extension at 72°C for 30 s; and a final extension step at 72°C for 10 min. The fragment length and concentration of PCR products were determined by 1% agarose gel electrophoresis (Guangzhou Ma Hao Biotechnology Co. Ltd., Guangzhou, China), followed by quantitative analysis of product concentrations using Gene Tools software (version 4.03.05.0, SynGene, Cambridge, UK). The mixed PCR products were purified using an EZNA gel extraction kit (Omega Bio‐Tek, Norcross, GA, USA) and eluted with TE buffer (REGAL, China) to recover the target DNA fragment. DNA libraries were created using the NEBNext Ultra Standard Program Specification DNA Library Preparation Kit (New England Biolabs, Ipswich, MA, USA). The DNA amplicon libraries were sequenced on the Hiseq. 2500 platform (Illumina, San Diego, CA, USA) using PE250 double‐end reads. The paired‐end raw reads were then trimmed using Trimmomatic software (V0.33, USADELLAB.org). Each paired‐end read was preprocessed and assembled using FLASH software (Efficient Length Adjustment of Short‐Read Sequences for Optimized Downstream Analysis, V1.2.11, https://ccb.jhu.edu/software/FLASH/) to perform read alignment and remove low‐quality sequences. The resulting high‐quality reads were then processed using Mothur software (V1.35.1, http://www.mothur.org) to filter the raw tag sequence, thereby generating clean tags.

### 2.5 Diversity Analysis

The sequence identity threshold was set at 97% using UPARSE software (version 10, http://www.drive5.com/usearch/). The representative sequences from the most frequently read operational taxonomic units (OTUs) were then assigned to annotation species at different taxonomic levels, and then linear discriminant analysis effect size (LEfSe) was analyzed using the Ribosomal Database Project classification based on QIIME. LEfSe was used to identify high‐dimensional biomarkers, providing insights into distinguishing genomic characteristics and their differential abundance across conditions. Bacterial diversity, richness, and abundance within the samples were evaluated using the Chao1, ACE, Simpson, and Shannon indices with a clustering threshold of 3% genetic distance. The diversity of the salivary microbiota in both the ESCC and control groups was assessed using weighted and unweighted UniFrac distances.

### 2.6 Cell Culture

The human ESCC cell line Eca109 (E109, Guangzhou, China) was cultured in RPMI 1640 (Gibco, Carlsbad, CA, USA) supplemented with 10% fetal bovine serum (Procell, Wuhan, China), 100 IU/mL penicillin, and 100 µg/mL streptomycin. Cells were cultured under a humidified atmosphere containing 5% CO_2_ at 37°C.

### 2.7 Microbial Strains and Cultivation Conditions

We cultured *F. nucleatum* (ATCC 25586) on blood agar plates (HuanKai, Guangzhou, China) for approximately 48–72 h. *P. gingivalis* (ATCC 33277) was cultivated on brain heart infusion (BHI) agar plates containing 10% defibrinated sheep blood and 0.5% vitamin K1 for 48 h. All bacterial cultures were incubated anaerobically at 37°C using AnaeroPack (Mitsubishi Gas Chemical Company, Tokyo, Japan).

### 2.8 Western Blot Analysis

The total cell protein was separated by 10% SDS‐PAGE and transferred to polyvinylidene fluoride membranes. The membranes were sealed with 5% skimmed milk powder in PBS containing 0.1% Tween‐20 and then probed with anti‐human PD‐L1 antibody (1:1000, 13684, Cell Signaling Technology, Danvers, MA, USA) or anti‐GAPDH antibody (1:5000, AP0063, Bioworld, Bloomington, MN, USA), followed by incubation with the secondary antibody (1:5000). We also use the ECL chemiluminescence system (Suzhou NCM Biotechnology Company, China) to detect proteins.

### 2.9 Flow Cytometry

To evaluate PD‐L1 expression on the cell membrane, E109 cells were preinfected with *P. gingivalis* (MOI = 1:100) for 6 h, followed by infection with *F. nucleatum* (MOI = 1:50) for another 48 h. Subsequently, the cells were resuspended in PBS and incubated with PE‐conjugated anti‐CD274 antibody (eBioscience, 12‐5983‐42, Thermo Fisher Scientific) or PE‐isotype control (eBioscience, 12‐4714‐82, Thermo Fisher Scientific) at 4°C for 40 min in the dark. Staining was performed on at least 10,000 cells per sample, and the samples were analyzed using a fluorescence‐activated cell sorting (FACS) system (CytoFLEX S, Beckman Coulter, Brea, CA, USA).

### 2.10 Statistical Analysis

R software (version 4.2.3, The R Project for Statistical Computing, Vienna, Austria) and RStudio were used for statistical analyses and graphing. The measurement data that conformed to a normal distribution were expressed as the mean ± standard deviation and analyzed using an independent‐samples *t*‐test. The abundance of microbial flora was expressed as a percentage and compared using the nonparametric Kruskal–Wallis test and Wilcoxon rank sum test. We use random forest models to evaluate the importance of biomarkers in distinguishing ESCC. *p* < 0.05 indicated statistical significance.

## 3 Results

### 3.1 *F. nucleatum* and *P. gingivalis* Can be Biomarkers for ESCC

**Figure 1:**
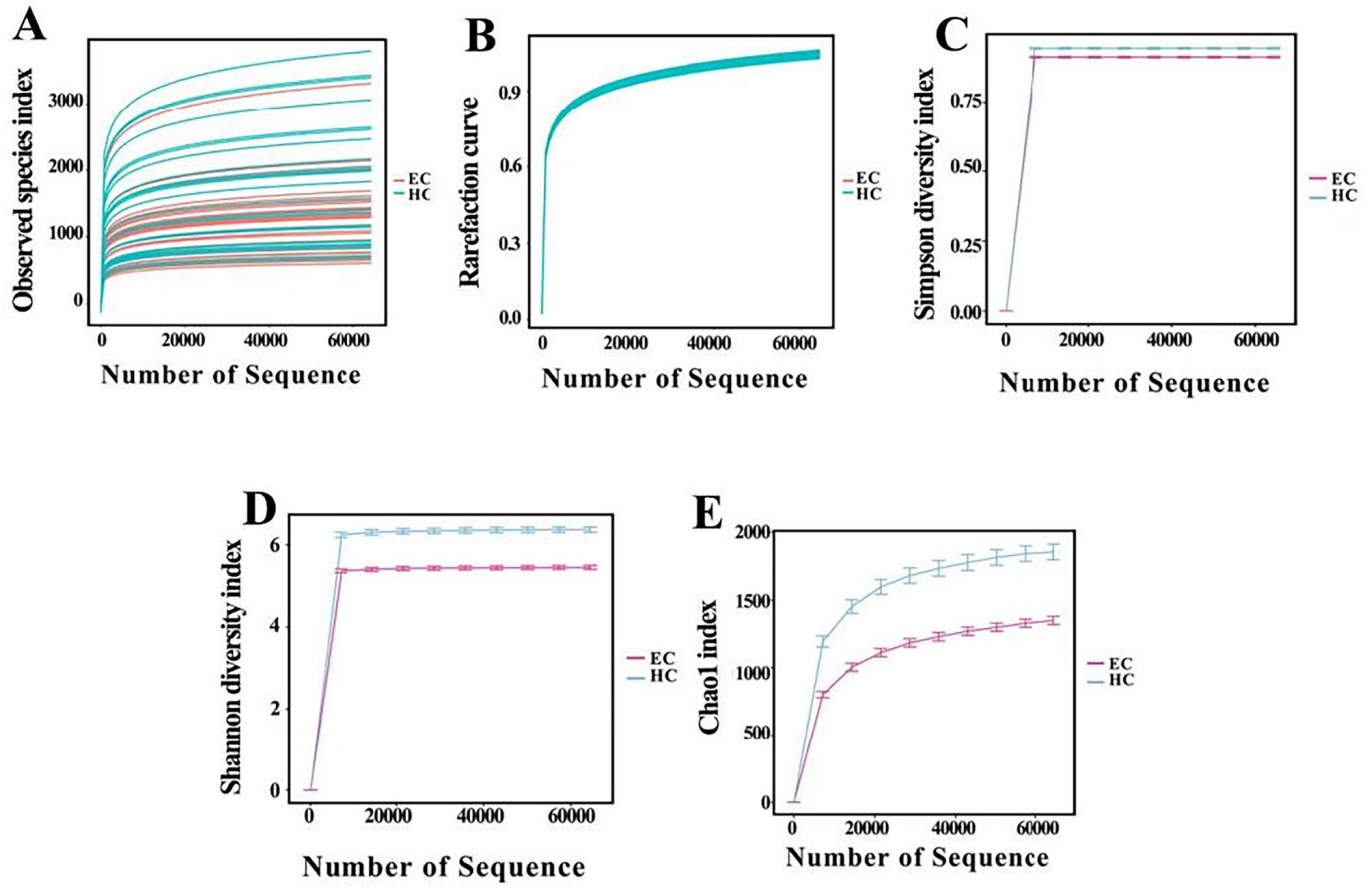
Alpha diversity analysis revealed significant variations in microbial abundance and diversity between the ESCC and control groups. EC, ESCC; HC, healthy control group. (A) Observed species index. (B) Rarefaction curve. (C) Simpson diversity index. (D) Shannon diversity index. (E) Chao1 index.

Following de‐noising, chimera removal, and quality trimming, 14,654,071 reads representing taxonomic units were analyzed. The sequencing reads were clustered into 10,3003 OTUs at a 97% similarity threshold, including 65,664 OTUs in the esophageal cancer group and 37,339 OTUs in the control group. We initially assessed the sequencing depth by generating and analyzing the sparsity curve for each sample (Figure 1A). The majority of the samples reached a sequencing plateau, indicating adequate sequencing. Furthermore, Good's coverage index exceeded 0.99 in both groups, suggesting that the obtained reads effectively captured the predominant bacterial diversity present in the study (Figure 1B).

Then, we used the Chao1, Shannon, and Simpson indices to compare the microbial diversity and abundance between patients with esophageal cancer and healthy controls. The Simpson diversity index was significantly higher in the ESCC group than in the control group (Figure 1C), whereas the Chao1 and Shannon diversity indices were significantly lower in the ESCC group than in the control group (Figure 1D,E).

**Figure 2:**
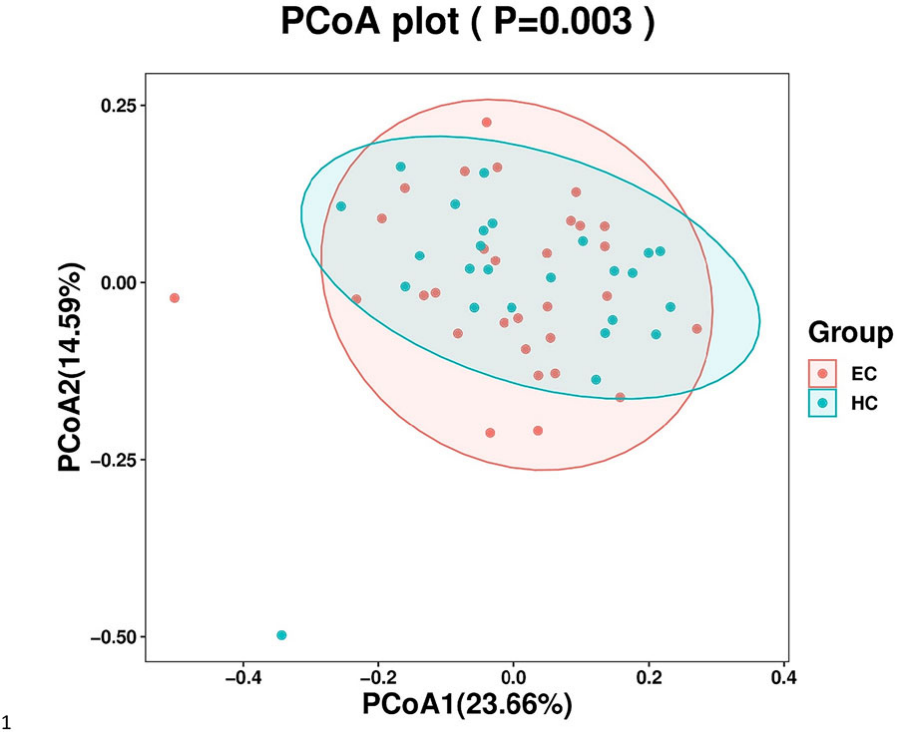
The PCoA plot of tissue samples from the ESCC and control groups.

We performed principal coordinate analysis (PCoA) to assess the overall structure of the salivary microbiota between the ESCC and control groups. PCoA revealed significant differences in overall microecology between the groups (*p* < 0.005, Figure 2).

**Figure 3:**
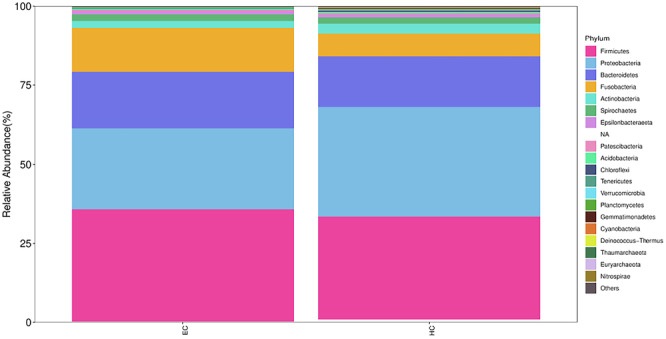
The relative abundance of microbes in the ESCC and control groups at the phylum level.

**Figure 4:**
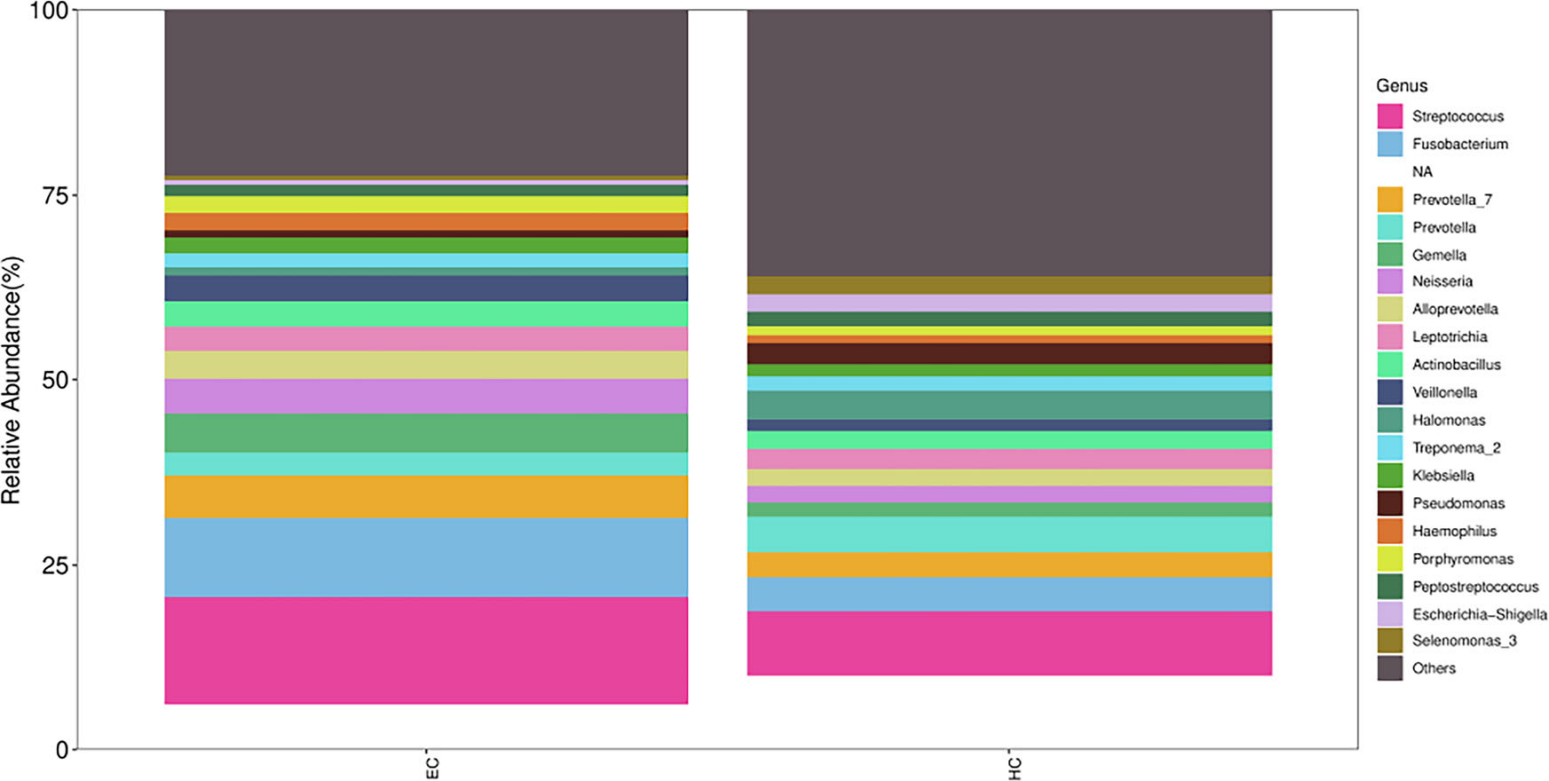
The relative abundance of microbes in the ESCC and control groups at the genus level.

We compared the relative abundance of microbes between patients with ESCC and healthy controls. More than 200 species were classified in the groups. The dominant bacteria at the phylum level (> 1% of total sequences in either group) included *Anaplasma*, *Aspergillus*, *Firmicutes, Clostridium*, *Actinomycetota, Aspergillus*, and *Spirochaetota*, which accounted for 99.53% and 99.54% of the salivary microbes in the ESCC and control groups, respectively (Figure 3). The predominant genera were similar between the groups. The most abundant genus in both groups was *Streptococcus*, followed by *Fusobacterium* (Figure 4).

**Figure 5:**
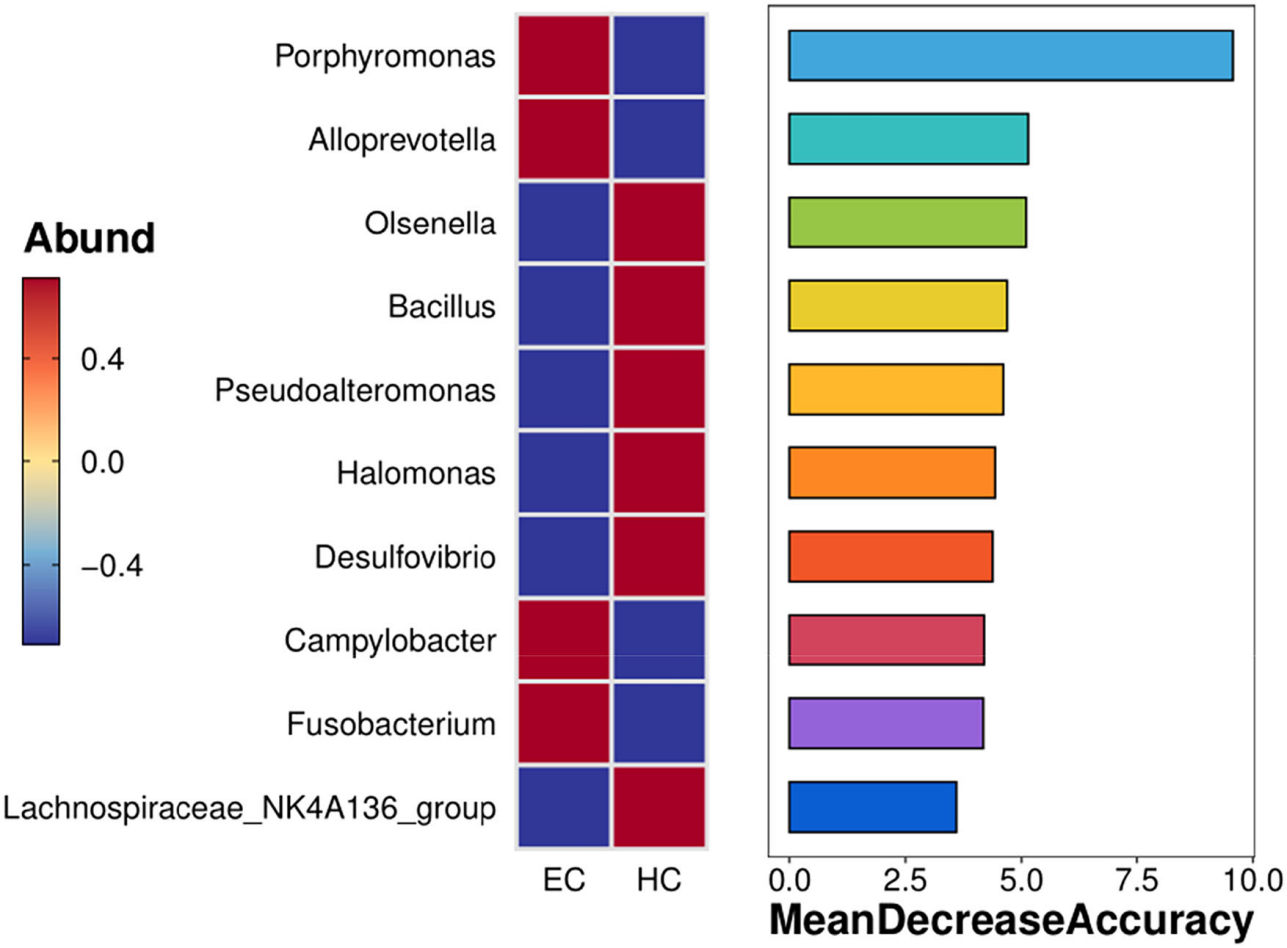
Ranking of the importance flora in distinguishing patients with esophageal cancer and healthy controls (random forest model). The figure illustrates the importance of different bacterial genera in distinguishing between healthy and cancerous tissues. Each bar represents a bacterial genus, with the length of the bar indicating its relative importance. The different colors correspond to different genera. *Porphyromonas gingivalis* and *Fusobacterium nucleatum* are highlighted as crucial in distinguishing between healthy and cancerous tissues, as indicated by their high feature importance scores.

Next, we used random forest to build models for the esophageal cancer intratissue flora and normal human intratissue flora (plotting the importance of variables), as presented in Figure 5. *F. nucleatum* and *P. gingivalis* played crucial roles in distinguishing healthy subjects from patients with esophageal cancer.

### 3.2 Quantitative Verification of the Target Bacteria in ESCC by qPCR

In total, 63 patients with ESCC and 17 controls were included in this study. The mean age of the ESCC group was 63.47 ± 7.70 years, versus 40.24 ± 13.64 years in the control group (*p* = 0.009). All participants were Chinese (Table 1).

**Table 1:** Baseline data of the enrolled subjects.

Variables	Total (*n* = 80)	ESCC group (*n* = 63)	HC group (*n* = 17)	Statistic	*p*
Sex, *n* (%)				*χ* ^2^ = 6.75	0.009
Female	17 (21.25)	9 (14.29)	8 (47.06)		
Male	63 (78.75)	54 (85.71)	9 (52.94)		
Smoking status, *n* (%)				*χ* ^2^ = 3.66	0.056
Former	40 (50.00)	35 (55.56)	5 (29.41)		
Never	40 (50.00)	28 (44.44)	12 (70.59)		
Alcoholic consumption, *n* (%)				*χ* ^2^ = 0.00	1.000
Former	20 (25.00)	16 (25.40)	4 (23.53)		
Never	60 (75.00)	47 (74.60)	13 (76.47)		

**Figure 6:**
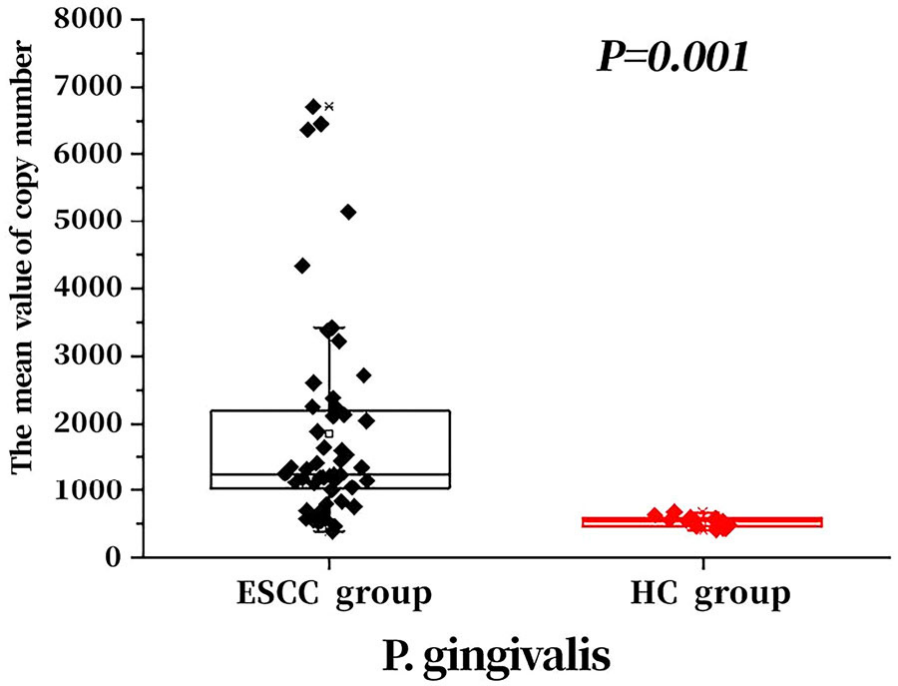
Comparison of *Porphyromonas gingivalis* copy numbers between the two groups.

**Figure 7:**
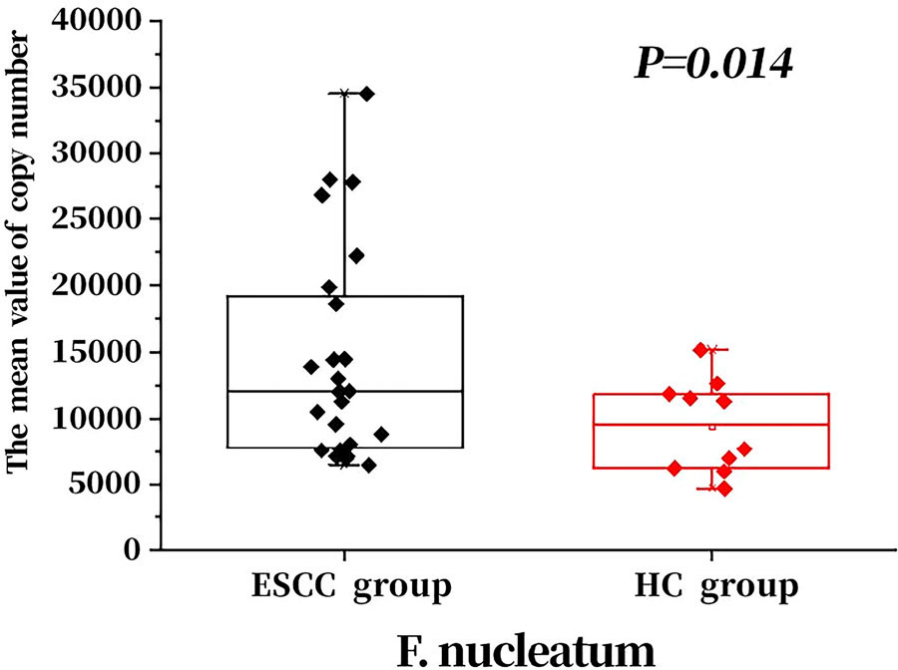
Comparison of *Fusobacterium nucleatum* copy numbers between the two groups.

Primers were designed according to the sequences of *P. gingivalis* and *F. nucleatum*, and the concentration of positive recombinant plasmids was determined by UV spectrophotometry. The plasmids were diluted 10‐fold, and standard curves were plotted with samples at concentrations of 1 × 10^−2^, 1 × 10^−3^, 1 × 10^−4^, 1 × 10^−5^, 1 × 10^−6^, and 1 × 10^−7^. The two bacteria were subjected to qPCR, and the results are presented in Figures 6 and 7.

### 3.3 *P. gingivalis* and *F. nucleatum* Were Related to PD‐L1 Expression in Esophageal Cancer Tissue

**Figure 8:**
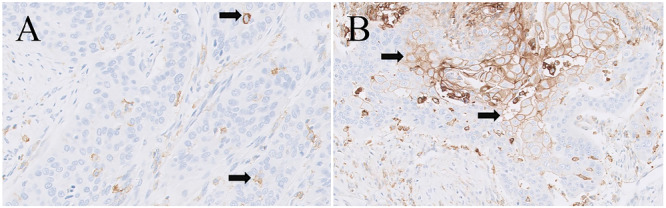
Negative and positive PD‐L1 staining in ESCC. (A) Negative (CPS = 7). (B) Positive (CPS = 45), CPS, combined positive score (×200).

**Figure 9:**
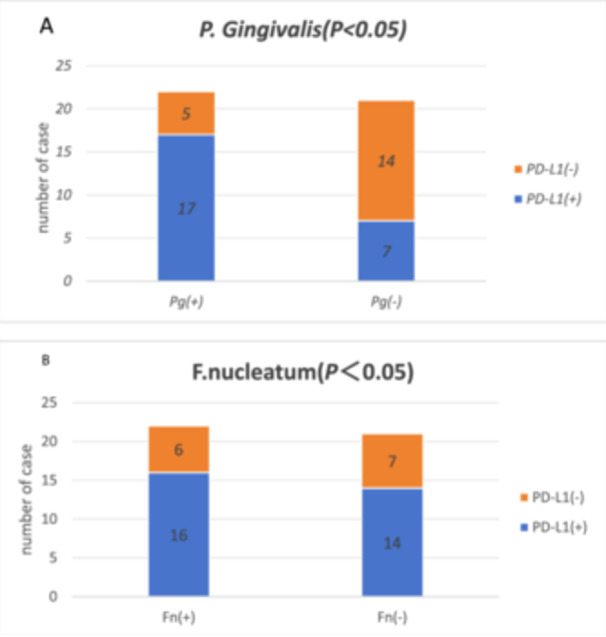
(A) Relationship between *Fusobacterium nucleatum* positivity and PD‐L1 expression. (B) Relationship between *Porphyromonas gingivalis* positivity and PD‐L1 expression.

Tissue sections of ESCC were immunohistochemically stained with PD‐L1 antibody, and PD‐L1 expression was scored using the PD‐L1 IHC 22C3 CPS scoring method to divide patients into the PD‐L1‐positive and PD‐L1‐negative groups. Finally, the relationships of *P. gingivalis* and *F. nucleatum* positivity in ESCC tissues and PD‐L1 positivity were compared by the chi‐squared test. Positivity for both *P. gingivalis* and *F. nucleatum* was related to PD‐L1 expression (both *p* < 0.05, Table 2 and Figures 8A,B and 9A,B).

**Table 2:** Detection of *Porphyromonas gingivalis*, *Fusobacterium nucleatum*, and PD‐L1 expression in cancer tissues.

	PD‐L1 (+)	PD‐L1 (−)	*χ* ^2^	*p*
*P*. *gingivalis* (+)	17	5	8.411	0.004
*P*. *gingivalis* (−)	7	14		
*F. nucleatum* (+)	16	6	6.702	0.010
*F. nucleatum* (−)	14	7		

### 3.4 *P. gingivalis* and *F. nucleatum* Promote PD‐L1 Expression in Esophageal Cancer Cells

**Figure 10:**
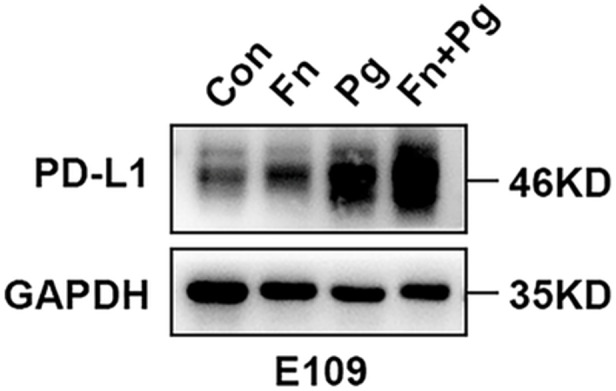
Immunoblot analysis of PD‐L1 expression in E109 esophageal cancer cells.

**Figure 11:**
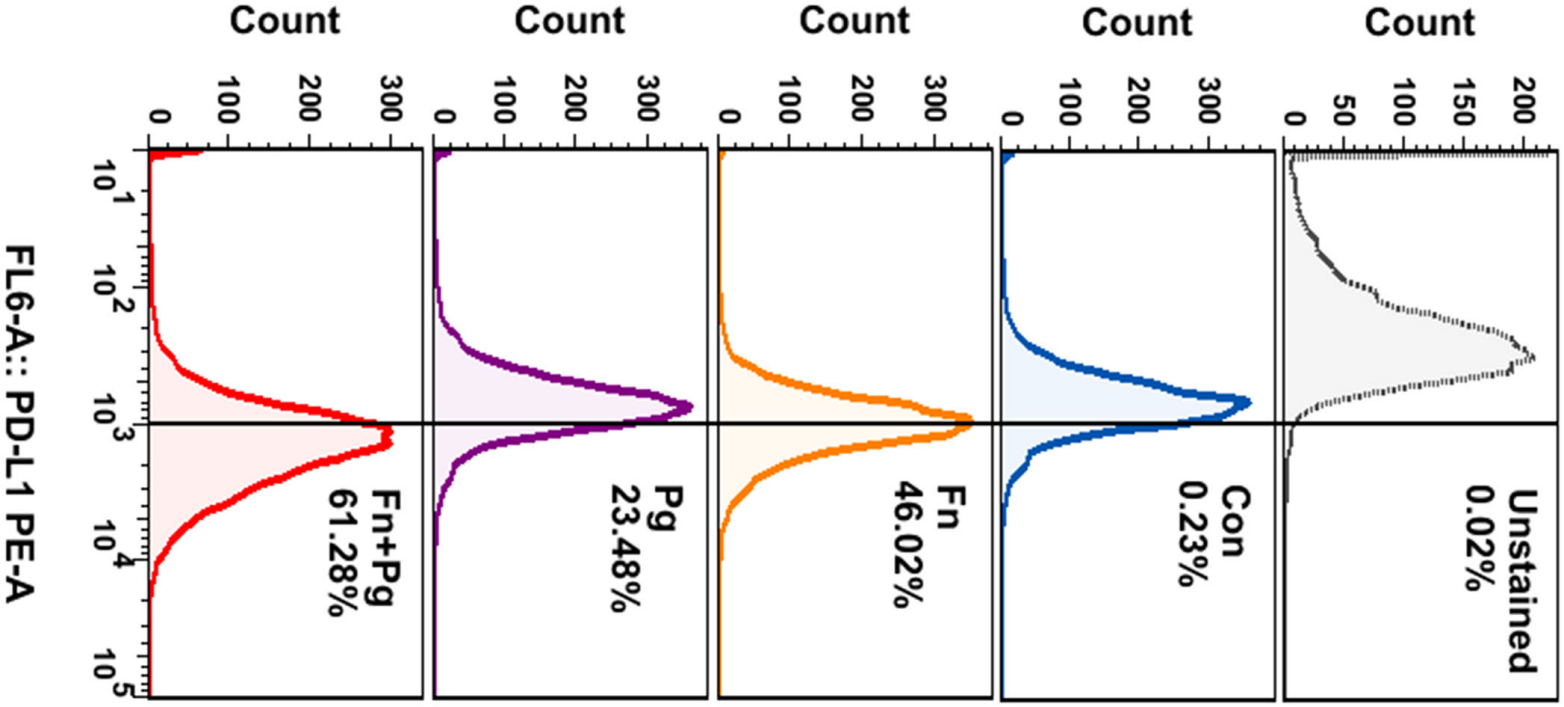
FACS analysis of PD‐L1 cell membrane expression in E109 esophageal cancer cells.

To investigate the effects of *P. gingivalis* and *F. nucleatum* infection on PD‐L1 protein expression, E109 cells were first infected with *P. gingivalis* (MOI = 1:100) for 6 h, followed by *F. nucleatum* (MOI = 1:50) for 48 h. As presented in Figure 10, infection with either bacterium alone increased PD‐L1 expression in E109 cells, and co‐infection synergistically increased PD‐L1 expression. In addition, flow cytometry revealed a significant increase in membrane PD‐L1 expression upon infection of E109 cells by *P. gingivalis* and/or *F. nucleatum* (Figure 11).

## 4 Discussion

Increasing evidence indicates that the microbiome influences the efficacy of immunotherapy in tumors for multiple reasons, including its effects on the human immune system and local immune microenvironment of the tumor. The role of PD‐L1/PD‐1‐mediated immune escape in the progression and recurrence of esophageal cancers has been extensively studied (Okadome et al. 2020; Ohigashi et al. 2005). Because PD‐L1 expression is one of the main factors influencing the efficacy of PD‐1/PD‐L1 inhibitors, patients with esophageal cancer are often treated with PD‐L1/PD‐L1 inhibitors according to their PD‐L1 status. Therefore, studying the factors affecting PD‐L1 expression might help guide the effective use of PD‐L1/PD‐1 inhibitors.

*P. gingivalis* and its membrane components can increase the expression of PD‐L1 in oral cancer cells (Groeger et al. 2011, 2017). In addition, *P. gingivalis* positivity in ESCC is positively correlated with tumor differentiation, lymph node metastasis, and TNM staging (Ren et al. 2023). Therefore, we speculate that *P. gingivalis* represents a noninvasive biomarker and therapeutic target for esophageal cancer.

Ren's study illustrated that *P. gingivalis* infection inhibited the cytotoxic activity of CD8^+^ T cells by increasing the phosphorylation of Akt and STAT3, resulting in PD‐L1 expression in dendritic cells (Ren et al. 2023). Our previous study revealed that *P. gingivalis* levels were significantly increased in saliva and tumor tissues in patients with ESCC compared with those in normal subjects. Further studies reported that *P. gingivalis* could activate the NF‐κB signaling pathway in esophageal cancer cells, thereby promoting esophageal cancer cell proliferation and motility. In addition, the expression of cyclin D1, MYC, MMP2, and molecules involved in the NF‐κB signaling pathway was increased in esophageal cancer cells, similarly as observed for PD‐L1 (Meng et al. 2019).

Recent studies revealed that *F. nucleatum* promotes the growth and migration of cancer cells by activating cancer‐promoting signaling pathways, thereby promoting the development and progression of gastrointestinal cancer (Casasanta et al. 2020; Yang et al. 2017; Nomoto et al. 2022). Furthermore, this microbe significantly affects patients' responses to chemotherapy and immunotherapy, highlighting its influence on clinical outcomes (Yang et al. 2017; Yu et al. 2017). Prior studies of esophageal cancer, including those by our group, detected high levels of *F. nucleatum* in tumor tissues and patient saliva, and *F. nucleatum* positivity was strongly related to esophageal cancer development (Li et al. 2021). Several studies confirmed that *F. nucleatum* is abundant in ESCC tissues, in which it drives cancer progression (Nomoto et al. 2022; Yamamura et al. 2016), and *F. nucleatum* infection indicates a high risk of ESCC metastasis (Li et al. 2021). A recent study by Zhang also found that *F. nucleatum* can survive and proliferate in ESCC cells, and *F. nucleatum* infection increased PD‐L1 expression in host cells, helping to prevent immune attack (Li et al. 2023). Therefore, *F. nucleatum* could be a potential treatment target for ESCC.

Substantial research has investigated the role of microbial communities in the occurrence and development of tumors. However, most of these studies focused on the role of a single bacterium, with few studies examining the potential combined effects of multiple bacteria on tumor microecology. Microorganisms generally have a combined effect on the human body and tumor tissues. For this reason, we investigated the relationships of multiple bacteria with the expression of immune molecules. *F. nucleatum* and *P. gingivalis* are among the most common bacteria in the microbiome in the healthy oral cavity and in periodontal lesions. These microorganisms have been linked to diseases such as cancer (Peng et al. 2022). Previous in vitro studies found that *F. nucleatum* co‐aggregates with *P. gingivalis* (Coppenhagen‐Glazer et al. 2015; Okuda et al. 2012), and these bacteria synergistically influence pathogenicity and biofilm formation (Horiuchi et al. 2020; Yamaguchi‐Kuroda et al. 2023). Feuille et al. found that virulence of *P. gingivalis* was increased by coculture with *F. nucleatum* (Feuille et al. 1996 *)*. In a mouse model of periodontitis‐related oral tumors caused by *F. nucleatum* and *P. gingivalis*, the bacteria interacted with the oral epithelium via TLRs to stimulate tumorigenesis. Furthermore, this effect was stimulated by enhanced signaling of the IL‐6/STAT3 axis (Gallimidi et al. 2015).

The coexistence of *F. nucleatum* and *P. gingivalis* has been reported in oral and colorectal cancer tissues (Zhang et al. 2019; Torralba et al. 2021; Guven et al. 2019; Gao et al. 2021). Clinical studies demonstrated that *F. nucleatum* and *P. gingivalis* are significantly enriched in tumor tissue, saliva, or stool samples from patients with cancer compared with the findings in healthy controls. Enrichment of the two bacteria could result in a worse prognosis, suggesting potential synergy between the bacteria. Nevertheless, the relationship of these two bacteria with the expression of the immune molecule PD‐L1 has not been investigated in tumors.

In this study, 16S rRNA sequencing demonstrated that *F. nucleatum* and *P. gingivalis* were enriched in ESCC, and this finding was subsequently validated by qPCR. Moreover, we proved by immunoblotting and flow cytometry that PD‐L1 expression was significantly increased in E109 cells following *F. nucleatum* and *P. gingivalis* co‐infection. Further, the presence of *F. nucleatum* and *P. gingivalis* in ESCC tissues was associated with increased PD‐L1 expression. These findings suggest that the presence of *F. nucleatum* and *P. gingivalis* could serve as an early warning sign of ESCC and as markers for predicting the efficacy of immunotherapy.

This study had some limitations. First, this experimental study only represents a preliminary investigation of the relationship between the microflora and PD‐L1 expression in esophageal cancer tissues, and further prospective research and in‐depth in vitro and in vivo experiments are necessary to explore the molecular mechanism of this relationship. Second, 16S rRNA gene sequencing can characterize the microbiota at the genus level, but it cannot identify microbes at the species level. Nevertheless, 16S rRNA gene sequencing has emerged as a pioneering approach in macrogenomic and other microbial studies because of its low cost and ease of sampling. Third, age significantly differed between the ESCC and healthy control groups, as patients with ESCC were substantially older than healthy controls. This asymmetry in the age distribution might have influenced the study findings.

In conclusion, this study identified elevated levels of both *P. gingivalis* and *F. nucleatum* in esophageal cancer tissues. Further experiments revealed that co‐infection of both bacteria in vitro resulted in significantly increased PDL1 expression. Additionally, positivity for *P. gingivalis* and *F. nucleatum* was associated with decreased PD‐L1 expression in ESCC tissues. It is postulated that targeting the actions of these two bacteria could increase the efficacy of immunotherapy.

## Author Contributions

**Xiayi Lin:** writing – original draft, writing – review and editing, conceptualization. **Xiaohui Chen:** methodology, data curation. **Mengyuan Chen:** investigation, validation. **Yiqiu Li:** methodology; data curation, resources. **Yu Deng:** conceptualization, investigation. **Bohong Xian:** writing – original draft, writing – review and editing. **Zijun Li:** conceptualization, methodology, funding acquisition.

## Ethics Statement

This study was conducted in accordance with ethical standards, and it was approved by the Ethics Committee of Guangdong Provincial People's Hospital (Ethical approval number is S2023‐576‐01). All participants provided written informed consent before their inclusion in the study, ensuring compliance with institutional and international guidelines on human research ethics.

## Conflicts of Interest

The authors declare no conflicts of interest.

## Data Availability

The data that support the findings of this study are available on request from the corresponding author. The data are not publicly available due to privacy or ethical restrictions.
